# Supplementary figures and images for: In silico prediction of blood cholesterol levels from genotype data
Source: PLoS One. 2020 Feb 10;15(2):e0227191. doi: 10.1371/journal.pone.0227191 (PMC7010235; doi:10.1371/journal.pone.0227191)

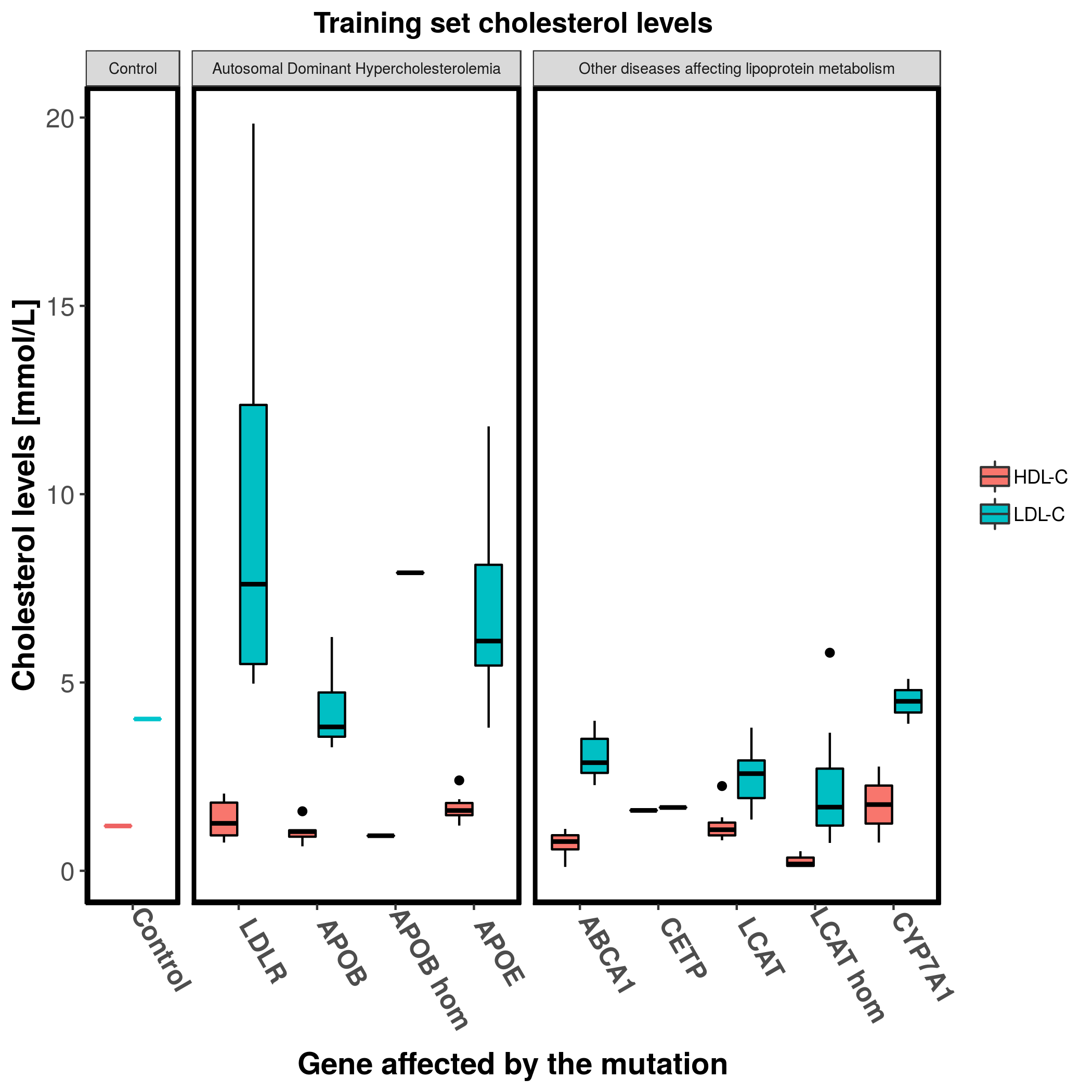

Supplement: S1 File — (ZIP) [file pone.0227191.s002.zip › S1_file/results/paper/Boxplot_chol_levels.tiff]

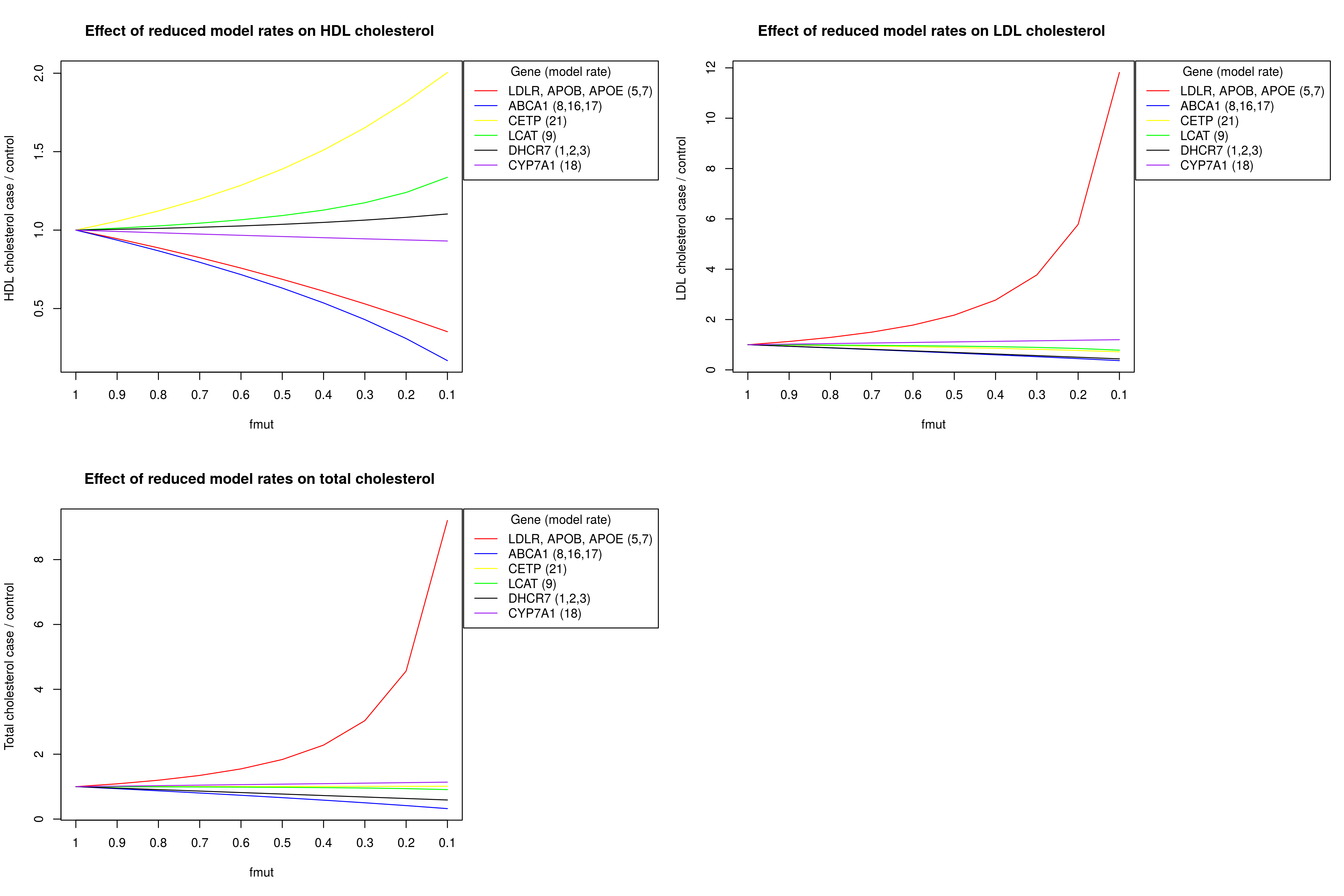

Supplement: S1 File — (ZIP) [file pone.0227191.s002.zip › S1_file/results/paper/HDL.levels.mutation.tiff]

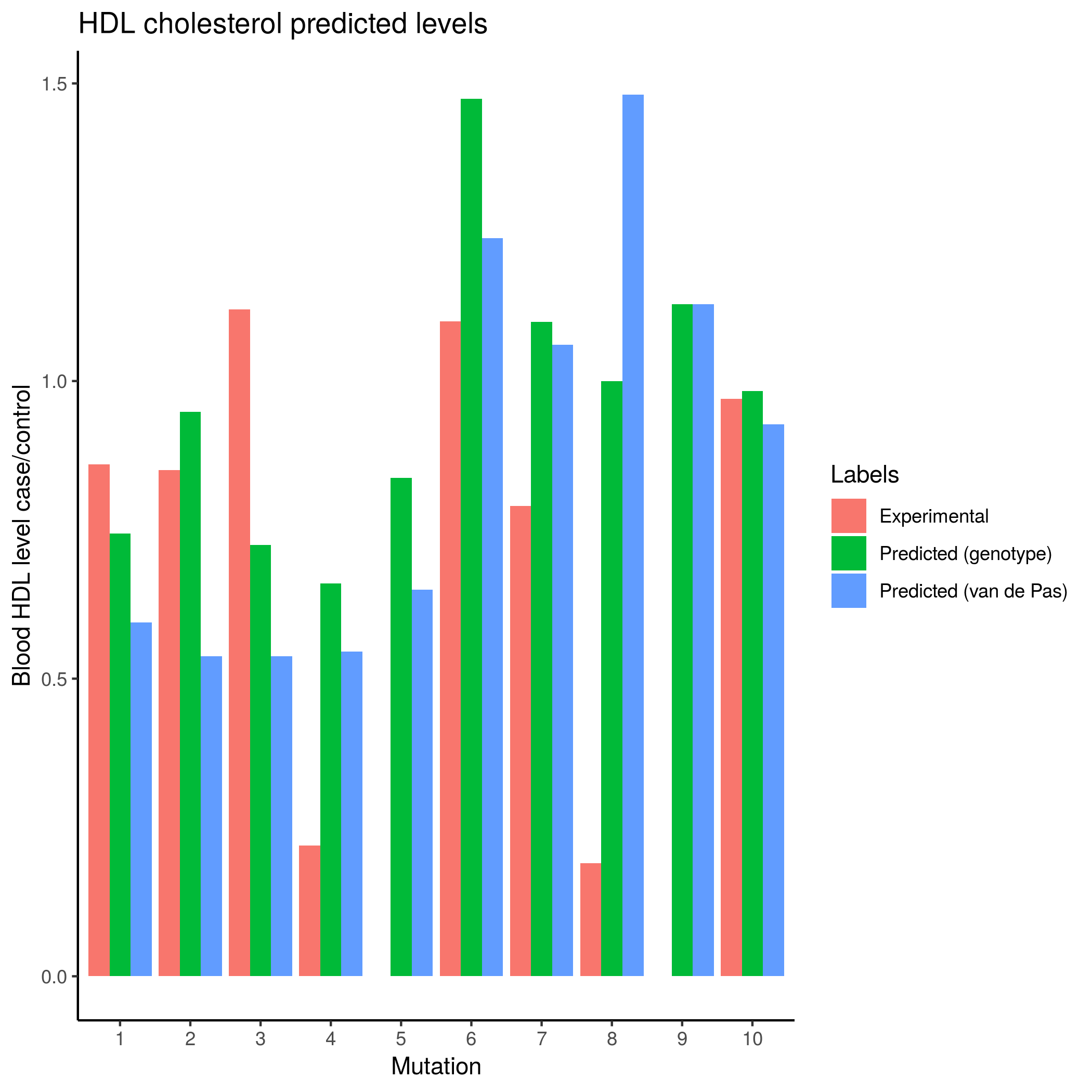

Supplement: S1 File — (ZIP) [file pone.0227191.s002.zip › S1_file/results/paper/HDL_chol_prediction.png]

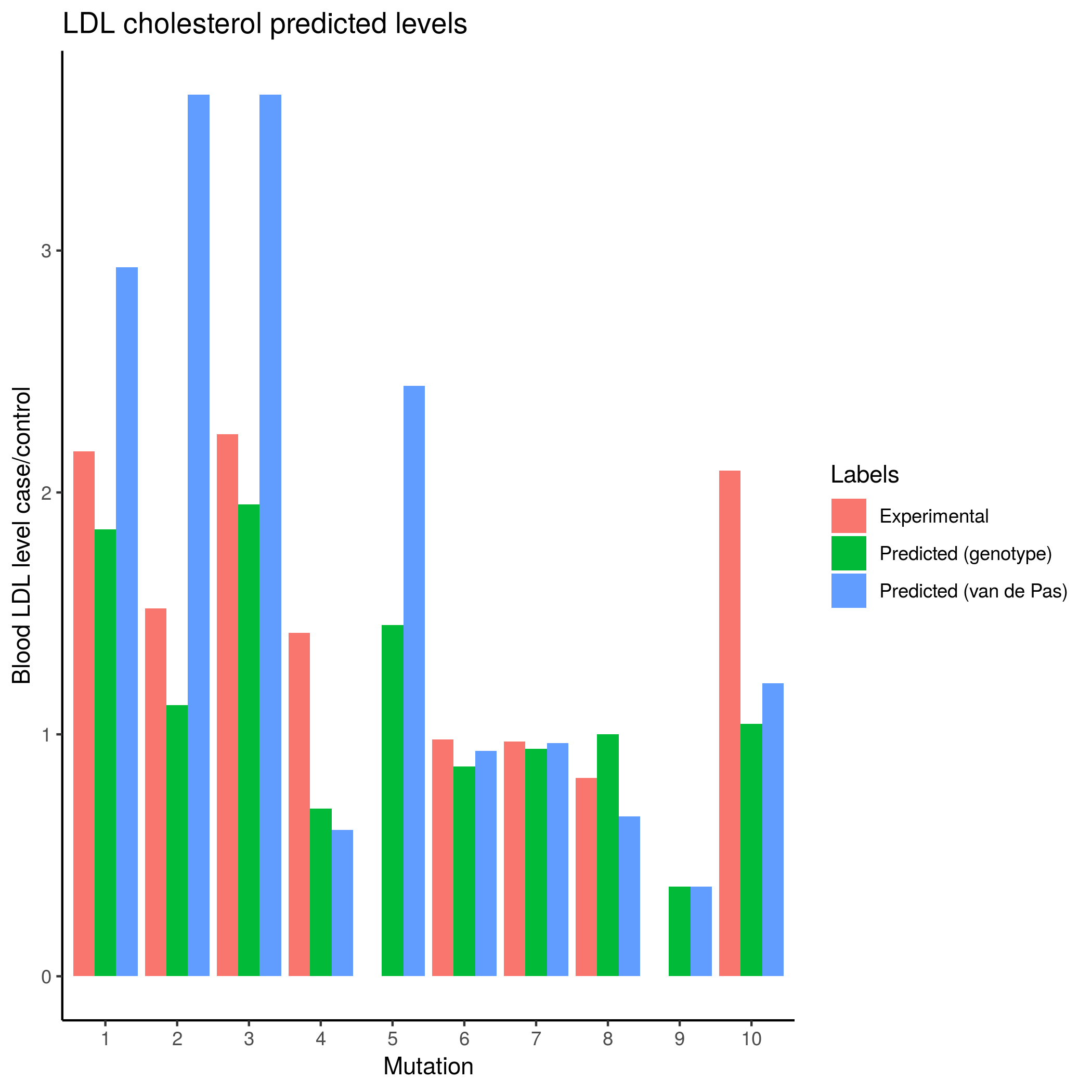

Supplement: S1 File — (ZIP) [file pone.0227191.s002.zip › S1_file/results/paper/LDL_chol_prediction.png]

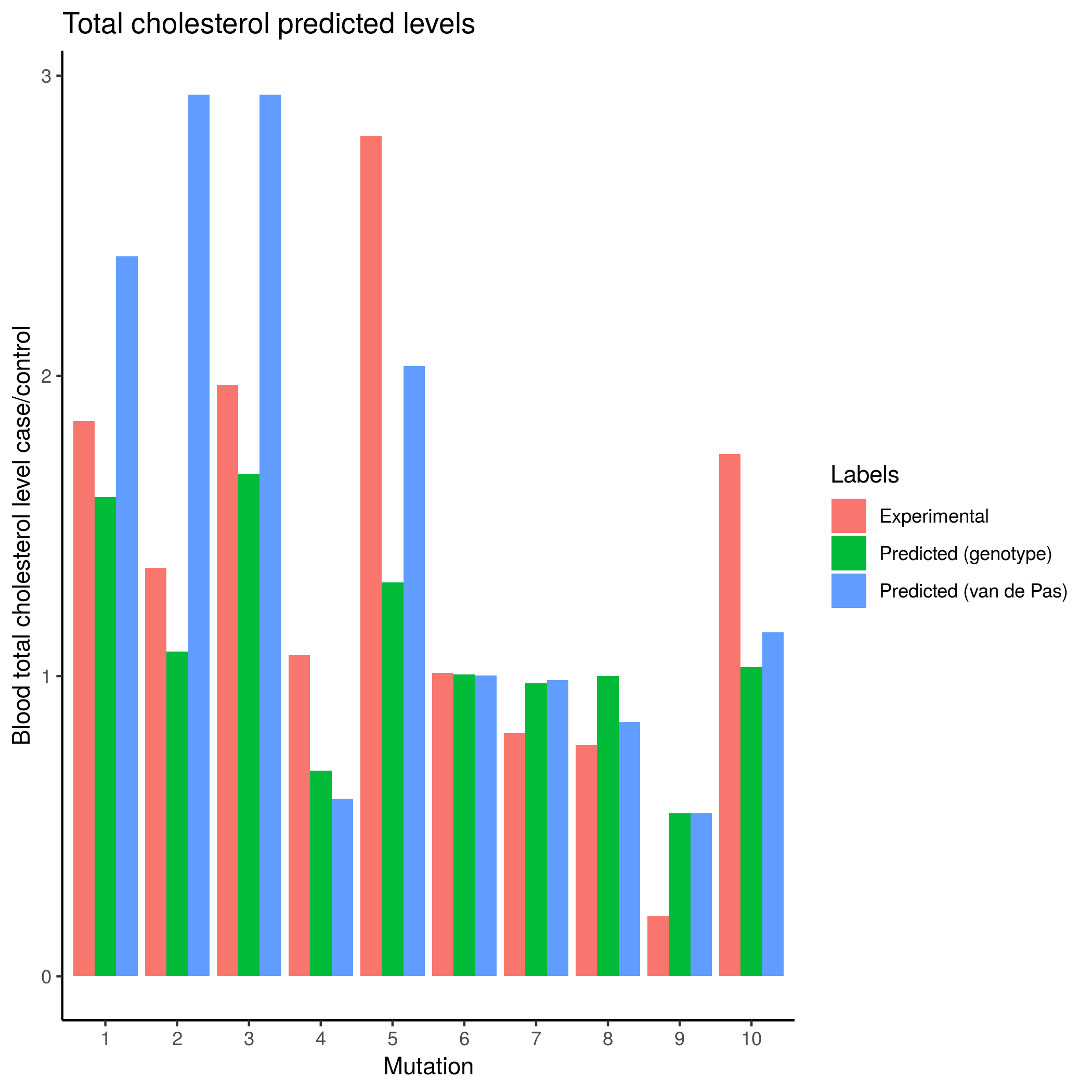

Supplement: S1 File — (ZIP) [file pone.0227191.s002.zip › S1_file/results/paper/Plot_total_chol.png]
